# Novel Naphthalene-Based Inhibitors of *Trypanosoma brucei* RNA Editing Ligase 1

**DOI:** 10.1371/journal.pntd.0000803

**Published:** 2010-08-24

**Authors:** Jacob D. Durrant, Laurence Hall, Robert V. Swift, Melissa Landon, Achim Schnaufer, Rommie E. Amaro

**Affiliations:** 1 Biomedical Sciences Program, University of California San Diego, La Jolla, California, United States of America; 2 Institute of Immunology and Infection Research, University of Edinburgh, Edinburgh, United Kingdom; 3 Department of Pharmaceutical Sciences, University of California Irvine, Irvine, California, United States of America; 4 Department of Biochemistry, Rosenstiel Basic Medical Sciences Center, Brandeis University, Waltham, Massachusetts, United States of America; 5 Department of Computer Science, University of California Irvine, Irvine, California, United States of America; Foundation for Innovative New Diagnostics (FIND), Switzerland

## Abstract

**Background:**

Neglected tropical diseases, including diseases caused by trypanosomatid parasites such as *Trypanosoma brucei*, cost tens of millions of disability-adjusted life-years annually. As the current treatments for African trypanosomiasis and other similar infections are limited, new therapeutics are urgently needed. RNA Editing Ligase 1 (REL1), a protein unique to trypanosomes and other kinetoplastids, was identified recently as a potential drug target.

**Methodology/Principal Findings:**

Motivated by the urgent need for novel trypanocidal therapeutics, we use an ensemble-based virtual-screening approach to discover new naphthalene-based *Tb*REL1 inhibitors. The predicted binding modes of the active compounds are evaluated within the context of the flexible receptor model and combined with computational fragment mapping to determine the most likely binding mechanisms. Ultimately, four new low-micromolar inhibitors are presented. Three of the four compounds may bind to a newly revealed cleft that represents a putative druggable site not evident in any crystal structure.

**Conclusions/Significance:**

Pending additional optimization, the compounds presented here may serve as precursors for future novel therapies useful in the fight against several trypanosomatid pathogens, including human African trypanosomiasis, a devastating disease that afflicts the vulnerable patient populations of sub-Saharan Africa.

## Author Summary

African sleeping sickness is a devastating disease that plagues sub-Saharan Africa. Neglected tropical diseases like African sleeping sickness cause significant death and suffering in the world's poorest countries. Current treatments for African sleeping sickness either have high costs, terrible side effects, or limited effectiveness. Consequently, new medicines are urgently needed. RNA editing ligase 1 is an important protein critical for the survival of *Trypanosoma brucei*, the unicellular parasite that causes African sleeping sickness. In this paper, we describe our recent efforts to use advanced computer techniques to identify chemicals predicted to prevent RNA editing ligase 1 from functioning properly. We subsequently tested our predicted chemicals and confirmed that a number of them inhibited the protein's function. Additionally, one of the chemicals was effective at stopping the growth of the parasite in culture. Although substantial work remains to be done in order to optimize these chemicals so they are effective and safe to use in human patients, the identification of these parasite-killing compounds is nevertheless a valuable step towards finding a better cure for this devastating disease.

## Introduction

Subspecies of *Trypanosoma brucei* (*T. brucei*) are the causative agents of human African trypanosomiasis (HAT, also known as African sleeping sickness) in sub-Saharan Africa. Neglected tropical diseases, including parasitic trypanosomal illnesses like HAT, Chagas disease, and leishmaniasis, are responsible for the loss of an estimated 56.6 million disability-adjusted life-years across several regions, particularly in the world's poorest countries [1]. Preventative measures such as vector control are effective at decreasing the incidence of HAT; however, given infection, the current treatment options are not suitable on the whole, particularly once *T. brucei* has infiltrated the central nervous system [2].

First-stage treatments include pentamidine and suramin, drugs developed more than half a century ago. Unfortunately, these drugs have severe side effects. Pentamidine is associated with hypoglycaemia and hypotension, while suramin is associated with anaphylactic shock, neurotoxic signs, severe cutaneous reactions, and renal failure [3]. The most common treatment for second-stage HAT is melarsoprol, a highly toxic drug with a 3%–10% fatality rate [4]. The danger of treatment is compounded by the emergence of melarsoprol-resistant parasites, particularly in central Africa [5]. Eflornithine, another HAT treatment, is less toxic but only effective against the *T. b. gambiense* subspecies; additionally, eflornithine is more costly to produce than melarsoprol [6]. Given the weaknesses of current treatments, new drugs are urgently needed.

Fortunately, recent studies of the trypanosomal editosome have revealed several new drug targets. In trypanosomatids, mitochondrial gene expression includes an extra RNA-editing step. As in other eukaryotes, mitochondrial DNA is transcribed into RNA. In trypanosomes and *Leishmania* parasites, however, a protein complex known as the editosome makes extensive uridylate (U) insertions and deletions following transcription, at times even doubling the length of the original RNA sequence [7]–[11]. After each cycle of U addition or deletion, a nick in the RNA remains; RNA editing ligase 1 (*Tb*REL1; TriTrypDB ID: Tb927.10.8210), an essential enzyme in trypanosomes [12], is one of two ATP-dependent editosome ligases responsible for religation. As the editosome is absent in humans, the proteins of this complex, including REL1, are potential drug targets in all trypanosomatid pathogens.

Recently Amaro et al. identified several novel *Tb*REL1 inhibitors. The relaxed complex scheme, a virtual-screening methodology that accounts for full protein flexibility [13], was used to identify five low-micromolar inhibitors from among the compounds of the National Cancer Institute Diversity Set I [14]. Unfortunately, these *Tb*REL1 inhibitors were ineffective against whole-cell *T. brucei*, perhaps in part because they are too hydrophilic to cross lipid membranes (unpublished work).

Motivated by both the urgent need for novel trypanocidal therapeutics as well as the success of virtual screening against *Tb*REL1 in the past, we here use the relaxed complex scheme to identify additional naphthalene-based inhibitors in hopes of finding compounds that can kill *T. brucei*. To this end, online databases of commercially available compounds were first searched for compounds similar to the inhibitors previously characterized. Following virtual screening, the most promising of these compounds were subsequently tested experimentally, revealing four novel *Tb*REL1 inhibitors with unique naphthalene-based scaffolds, two of which have ALogP values that suggest reasonable lipophilicity. Analyses of the predicted binding modes of these active compounds, performed using an ensemble-based approach and coupled with computational fragment mapping experiments, suggest that receptor flexibility may play an important role in ligand binding.

## Methods

### Online Similarity Search

**Figure 1:**
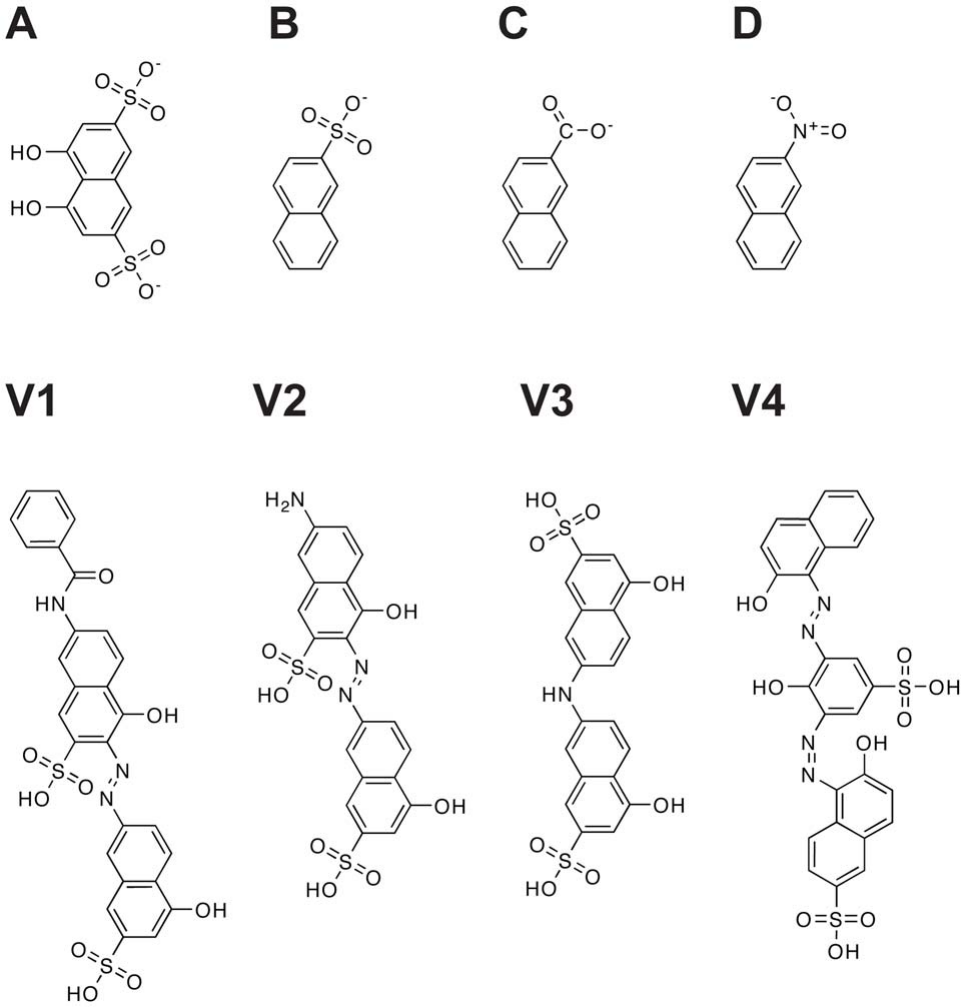
Substructures and novel inhibitors. A) The core 4,5-dihydroxynaphthalene-2,7-disulfonate scaffold of three previously identified *Tb*REL1 inhibitors. B) Naphthalene-2-sulfonic acid. C) 2-naphthoic acid. D) 2-nitronaphthalene. V1-V4) The four low-micromolar inhibitors identified in the current work.

To generate a library of compounds similar to the *Tb*REL1 inhibitors characterized previously [14], we performed online substructure searches of several databases of commercially available compounds, including Hit2Lead (Hit2Lead.com, ChemBridge), the NCI/DTP Open Chemical Repository (dtp.cancer.gov), Sigma-Aldrich (sigmaaldrich.com), and ZINC [15]. Searches were performed using three structures similar to the core naphthalene scaffolds of known inhibitors: naphthalene-2-sulfonic acid, 2-naphthoic acid, and 2-nitronaphthalene (Figure 1).

### Initial Docking against the Crystal Structure

The compounds identified *via* online substructure searches were each docked into a 1.20-Å resolution crystal structure of the *Tb*REL1 catalytic domain (PDB ID: 1XDN) [16] using AutoDock 4 [17]. Ligand files were processed with AutoDockTools 1.4.5 to merge nonpolar hydrogens with parent heteroatoms and to assign Gasteiger charges. AutoGrid affinity grids contained 86×72×78 points spaced 0.375 Å apart, centered on the *Tb*REL1 active site, the ATP-binding pocket. Grid files were created for the following ligand atom types: A (aromatic carbon), C, F, I, N, NA (hydrogen-bond accepting N), Cl, OA (hydrogen-bond accepting O), P, S, SA (hydrogen-bond accepting S), Br, HD (hydrogen-bond hydrogen), and e (electrostatic).

AutoDock parameters similar to those published previously by Amaro et al. [14] were used: population size 200; 5,000,000 evaluations; 27,000 generations; 100 runs; and cluster tolerance of 2.0 Å. All other AutoDock parameters were set to the default values. The correct docked pose was judged to be the lowest-energy pose of the most populated cluster.

### MD Simulation

With the intent of rescoring the top hits from the initial crystal-structure screen in a way that accounts for full protein flexibility, we drew upon a previous study of *Tb*REL1 molecular motions [18]. In brief, molecular dynamics (MD) simulations of *Tb*REL1 were performed using NAMD 2.6 [19]. Four hundred receptor conformations were extracted from the MD simulations, one every 50 ps. QR factorization [20] was used to eliminate conformational redundancy, thereby reducing the number of representative structures from 400 to 33 [14]. These 33 *Tb*REL1 structures are said to constitute an *ensemble* representative of the many protein conformations sampled during the MD simulation.

### Ensemble-Based Virtual Screening with the Relaxed Complex Scheme

The relaxed complex scheme (RCS) was subsequently used to rescore the top compounds from the initial crystal-structure screen [13]. AutoDock was used to dock each of the top inhibitors into the 33 protein conformations of the receptor ensemble using the same docking parameters described above. The ensemble-average binding energy of each ligand was computed by taking the simple mean, and the ligands with the best mean predicted binding energy were subsequently tested experimentally.

### RMSD Clustering

To partition the ATP-bound trajectory [18] into a set of structures representing regions of decreasing conformational population density, RMSD clustering, distinct from the QR factorization described above, was performed [21]–[23] as implemented in the rmsdmat2 and cluster2 programs of the GROMOS++ analysis software [24]. Four hundred receptor conformations were extracted from the 20 ns ATP-bound MD trajectory, one every 50 ps. Clustering was performed on a subset of 24 residues that line the ATP binding cleft: 87–90, 155–162, 207–209, 283–287, and 305–308. These residues constitute the 5 conserved motifs of the nucleotidyltransferase superfamily [25], [26] to which *Tb*REL1 belongs. The trajectory frames were first aligned by minimizing the RMSD between the alpha carbons of the 24-residue subset of each frame and the corresponding alpha carbons of the first frame. This least-squares alignment removed external translational and rotational motion so that subsequent RMSD calculations could focus on the internal conformational variability of the 24-residue subset. After varying the RMSD similarity criterion from 0.06 to 0.12 Å, a value of 0.085 Å was chosen, as this cutoff produced 8 clusters of protein conformations. The three most populated clusters comprised 93.5% of the trajectory.

### Computational Fragment Mapping

Computational fragment mapping (FTMap, http://ftmap.bu.edu) was utilized to identify druggable regions on the surface of *Tb*REL1. The FTMap algorithm [27] determines the energetically favorable binding regions of sixteen fragments along a protein surface (Figure S1) *via* the following steps: (1) rigid body docking of fragments using a fast Fourier transform approach, (2) minimization and rescoring of fragment-protein complexes, (3) clustering and ranking of low-energy fragment-protein complexes, and (4) determination of consensus sites. Consensus sites are regions of the protein surface where low-energy fragment clusters of multiple fragment types co-localize; in previous studies using FTMap and its predecessor CSMap [28], highly populated consensus sites were shown to correlate strongly with ligand binding hot spots identified *via* biophysical methods [27], [29], [30].

### Experimental Validation

The top ranked compounds from the relaxed complex screen were obtained for testing in experimental assays. Compounds were provided by the Developmental Therapeutic Program at the National Cancer Institutes (NCI) of Health, Hit2Lead.com, and Sigma-Aldrich (Table S1). Compounds V1, V2, and V3 (Figure 1) were provided by the NCI, and compound V4 was purchased from Sigma. All compounds were dissolved in DMSO or DMSO/H_2_O.

The protocols for recombinant *Tb*REL1 expression, purification, and assaying have been described previously [14]. In brief, recombinant full-length *Tb*REL1 was expressed in Sf9 insect cells after infection with recombinant baculovirus and purified *via* a C-terminal tandem affinity purification (TAP) tag. To measure enzyme inhibition, 0.1 pmol *Tb*REL1 was incubated with 1.8 µCi (30 nM) [α-^32^P]ATP in assay buffer (25 mM KCl, 12.5 mM HEPES pH 7.9, 5 mM Mg acetate, 0. 25 mM DTT, 0.1% Triton X-100) for 5 min at room temperature and in the presence of varying concentrations of the potential inhibitors. The extent of protein adenylylation (and therefore competition with ATP for binding to the active site) was subsequently measured by SDS/PAGE and phosphorimaging. All reactions were done in at least triplicate, and IC_50_ values were calculated using the GraphPad Prism 5 software.

### *T. brucei* Viability Assay

The effect of the identified REL1 inhibitors on parasite growth was determined using the Alamar Blue assay, essentially as described by Räz et al. [31]. Briefly, *T. brucei brucei* cells (strain s427) were seeded in 96-well plates at a density of 1×10^4^ cells per ml in a volume of 200 µl, in the presence of varying concentrations of predicted inhibitors or DMSO alone. After 48 hours, 20 µl Alamar Blue (Invitrogen) were added to the cells and incubation continued for an additional 24 hours. Absorbances at 540 and 595 nm were measured using an ELx808 Microplate Reader (BioTek), and EC_50_ values were calculated using the GraphPad Prism 5 software.

## Results and Discussion

RNA editing ligase 1 (REL1) is a key component of the trypanosomatid editosome. In trypanosomatid parasites (i.e. species of *Trypanosoma* and *Leishmania*), mitochondrial mRNA requires editing following transcription; after each round of U addition or deletion, REL1 and the related protein REL2 religate the RNA in an ATP-dependent reaction. REL1 is a noteworthy drug target because it is required for the survival of *T. brucei* [12], [32] and presumably other trypanosomatids as well. Additionally, no close human homologues have been identified [14]. The heavy disease burdens caused by human African trypanosomiasis (HAT), Chagas disease, and leishmaniasis, as well as the urgent need for novel trypanocidal therapeutics and the success of virtual screening against *Tb*REL1 in the past, have motivated the current work, wherein we identify novel *Tb*REL1 inhibitors with naphthalene-based scaffolds.

### Similarity Search

**Figure 2:**
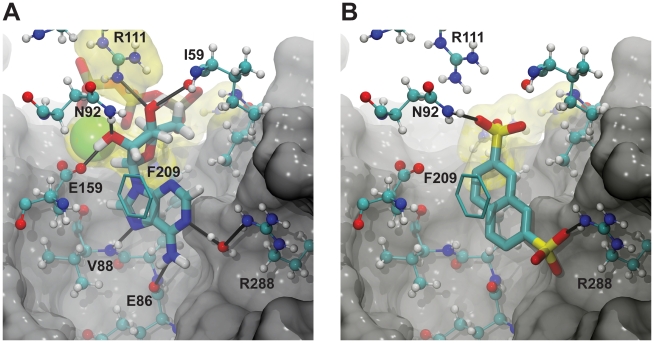
*Tb*REL1 binding. Solid black lines represent hydrogen bonds. Positively charged residues and ions (Mg^2+^) at the active-site periphery are highlighted in yellow. The carbons of the F209 phenyl ring are shown in licorice. Portions of the protein are not visualized to improve clarity. A) ATP binding. B) The predicted binding pose of naphthalene-2,7-disulfonate.

Previously, Amaro et. al identified several micromolar inhibitors of *Tb*REL1 [14]. The top three inhibitors identified were all based on a naphthalene-2,7-disulfonate (NDS) scaffold. *In silico* docking provides insight into why this scaffold is amenable to *Tb*REL1 inhibition (Figure 2). Similar to the adenine moiety of ATP (the native co-factor), the NDS naphthalene group is able to form π-π stacking interactions with F209. Additionally, one of the negatively charged NDS sulfonate groups interacts electrostatically with the positively charged R111 guanidino group at the active-site periphery; R111 also participates in electrostatic and hydrogen-bond interactions with the ATP polyphosphate tail. A hydrogen bond is formed between NDS and N92, similar to the hydrogen bond formed with the O2' oxygen atom of the ATP ribose. Finally, docking suggests that the second of the two NDS sulfonate groups is buried deep within the binding pocket, displacing a water molecule that normally mediates a hydrogen-bond network between the ATP adenine N1 atom and R288. This water displacement allows the sulfonate group to interact with the charged R288 residue directly.

Unfortunately, these previously identified *Tb*REL1 inhibitors were ineffective against whole-cell *T. brucei*, likely because they are too hydrophilic to cross lipid membranes (A. Schnaufer, unpublished work). Interestingly, these compounds show similarities to the anti-trypanosomal drug suramin, which, although much larger, also has a negatively charged polysulfonated naphtyl group [14]. How suramin enters the cell is unclear, but both fluid-phase endocytosis and receptor-mediated uptake have been suggested [33], [34]. Suramin both binds various serum proteins, which may facilitate uptake by the trypanosome cell [34], –and inhibits a considerable number of enzymes, including dehydrogenases and kinases in various organisms and glycolytic enzymes in *T. brucei* [35]. This promiscuous binding may be in part attributable to the negatively charged sulfonate groups [35]. Additionally, the hydrophilicity these sulfonates impart likely impedes both suramin and the previously identified REL1 inhibitors from passively crossing inner cellular membranes to reach organellar targets such as mitochondrial proteins.

In an attempt to identify additional naphthalene-based *Tb*REL1 inhibitors with improved pharmacological properties, we searched several online databases of commercially available compounds for similar structures: naphthalene-2-sulfonic acid, 2-naphthoic acid, and 2-nitronaphthalene (Figure 1). These searches identified 588 compounds: 61 compounds from Hit2Lead (Hit2Lead.com, ChemBridge), 394 from the NCI/DTP Open Chemical Repository (dtp.cancer.gov), 87 from Sigma-Aldrich (sigmaaldrich.com), and 46 from ZINC [15]. In all, the search identified 376 naphthalene-2-sulfonic acid compounds, 130 2-naphthoic acid compounds, and 85 2-nitronaphthalene compounds.

### Virtual Screen

Given its previous successful identification of *Tb*REL1 inhibitors, AutoDock 4.0 was utilized for docking. Although the AutoDock scoring function sacrifices accuracy for speed as compared to more rigorous methodologies such as thermodynamic integration [17], [36], single-step perturbation [37], and free energy perturbation [38], AutoDock performs well [39] when compared to other docking programs such as DOCK [40], FlexX [41], and GOLD [42].

The 588 compounds identified through online substructure searches were first docked into a 1.20-Å resolution crystal structure of the catalytic domain of *Tb*REL1 [16]. AutoDock placed 14% of the naphthalene compounds in the expected pose (26% of the 2-naphthoic acid compounds, 10% of the naphthalene-2-sulfonic acid compounds, and 8% of the 2-nitronaphthalene compounds), with the naphthalene portion of the ligand buried deep in the ATP-binding pocket and the electronegative group at the two position either interacting with R288 or with R111 at the active-site periphery.

The preliminary docking to the *Tb*REL1 crystal structure, while useful for eliminating those structures that were grossly incompatible with the *Tb*REL1 active site, did not account for full protein flexibility. Aside from the inaccuracies inherent in docking scoring functions themselves, docking accuracy decreases further when protein and/or ligand flexibility are ignored. When a ligand approaches a protein receptor in solution, it does not encounter a single static protein conformation, but rather an ensemble of many different conformations. Often, a given ligand may only bind to a certain subset of all protein conformations sampled, depending in part on the varied side-chain positions of active-site residues. When multiple protein conformations are incorporated into a virtual-screening protocol, the hit rate can drastically improve; ligands that do not bind to the crystal structure may bind to other related protein conformations. Screening against these other conformations in principle reduces the false negative rate.

Of the top-ranked 100 binders from preliminary crystal-structure screens, 45 shared significant structural similarity with the most potent compound identified previously by Amaro et al. [14]. In order to account for full protein-receptor flexibility, these 45 compounds, roughly corresponding to the top 7.5% of the library, were docked into 33 protein receptor conformations extracted from a MD simulation of *Tb*REL1 [14] using QR factorization [20]. The 45 ligands were then reranked by their respective ensemble-average scores, and 12 of the top compounds (Table S1) were subsequently tested experimentally.

### Experimental Results

Prior to RNA ligation, a key *Tb*REL1 lysine must first be adenylylated. To measure the inhibition of this first step of the reaction pathway, the formation of *Tb*REL1-[^32^P]AMP was monitored *via* SDS/PAGE and autoradiography in the presence of predicted inhibitor. Triton X-100 (0.1%) was added in order to prevent aggregate-based inhibition. Four compounds, V1, (E)-7-benzamido-4-hydroxy-3-((5-hydroxy-7-sulfonaphthalen-2-yl)diazenyl)naphthalene-2-sulfonic acid; V2, (E)-7-amino-4-hydroxy-3-((5-hydroxy-7-sulfonaphthalen-2-yl)diazenyl)naphthalene-2-sulfonic acid; V3 (Di-J acid); and V4 (Mordant Black 25), inhibited *Tb*REL1 activity with IC_50_ values of 2.16±1.20 µM, 1.53±1.17 µM, 8.36±1.71 µM, and 1.59±1.1 µM, respectively (Table 1, Figure 1). Additional information about the predicted binding poses of these four validated inhibitors can be found in Table S1. An additional four compounds inhibited *Tb*REL1 adenylylation with IC_50_ values between 10 and 100 µM; the exact values in these cases were not determined (Table S1). All other compounds did not show significant inhibition at 100 µM.

**Table 1:** A summary of the computational and experimental results.

	V1	V2	V3	V4
IC_50_	2.16±1.20	1.53±1.17	8.36±1.71	1.59±1.10
EC_50_	>100	>100	>100	2.16±0.25
AutoDock_Crystal_	−11.8	−11.3	−10.7	−11.8
Rank_Crystal_	11	20	31	25
AutoDock_Ensemble_	−11.9±1.4	−11.9±1.4	−10.2±1.0	−12.8±1.6
Rank_Ensemble_	4	3	12	1
% Expected Pose	33%	33%	24%	18%
AutoDock_Ensemble/Expected_	−13.3±1.2	−12.6±1.1	−11.6±0.5	−14.9±0.6
AutoDock_Ensemble/Unexpected_	−11.2±0.9	−11.6±1.4	−9.8±0.6	−12.3±1.4
AutoDock_Ensemble/Best_	−15.3	−13.9	−12.1	−15.6

IC_50_ and EC_50_ are measures (in µM) of the inhibition of REL1 activity and parasite growth, respectively; AutoDock_Crystal_ is the predicted binding energy, in kcal/mol, to the crystal structure; Rank_Crystal_ is the rank of the ligand when all 588 compounds are ordered by their respective AutoDock_Crystal_ values; AutoDock_Ensemble_ is the average predicted binding energy to the 33 representative protein-receptor conformations obtained *via* QR factorization, plus or minus the standard deviation; Rank_Ensemble_ is the rank of the ligand when the top 45 compounds are ordered by their respective AutoDock_Ensemble_ values; % Expected Pose is the percentage of the 33 representative protein structures amenable to deep-pocket binding, in which the naphthalene core is docked deep into the binding pocket; AutoDock_Ensemble/Expected_ is the average predicted binding energy when only those members of the ensemble amenable to deep-pocket binding are considered; AutoDock_Ensemble/Unexpected_ is the average predicted binding energy when only the remaining members of the ensemble are considered; and AutoDock_Ensemble/Best_ is the predicted binding energy of the ligand to the “optimal protein conformation” from the ensemble. All predicted energies are in kcal/mol.

### Binding to the Crystal-Structure Protein Conformation May Be Suboptimal

Interestingly, the crystal-structure protein conformation used for the initial docking is likely itself suboptimal for the binding of the four inhibitors identified, as evidenced by the improvement in rank when an ensemble-average AutoDock score was used (Rank_Ensemble_) instead of the crystal-structure score (Rank_Crystal_, Table 1). In fact, only one of the four compounds, V1, scored in the top twelve when all 588 compounds were docked into the crystal structure alone. V2, V3, and V4, which ranked 20^th^, 31^st^, and 25^th^ against the crystal structure, respectively, may not have been tested had the ligand set not been reranked by an ensemble-average AutoDock score. A direct comparison of the predicted binding energy of the four indentified inhibitors docked into the crystal structure (AutoDock_Crystal_) and docked into the optimal protein conformation from the ensemble (AutoDock_Ensemble/Best_) likewise demonstrates the importance of accounting for full protein flexibility; in all four cases, predicted energies of binding improved several kcal per mol when the optimal structure was used rather than the crystal structure (Table 1).

**Figure 3:**
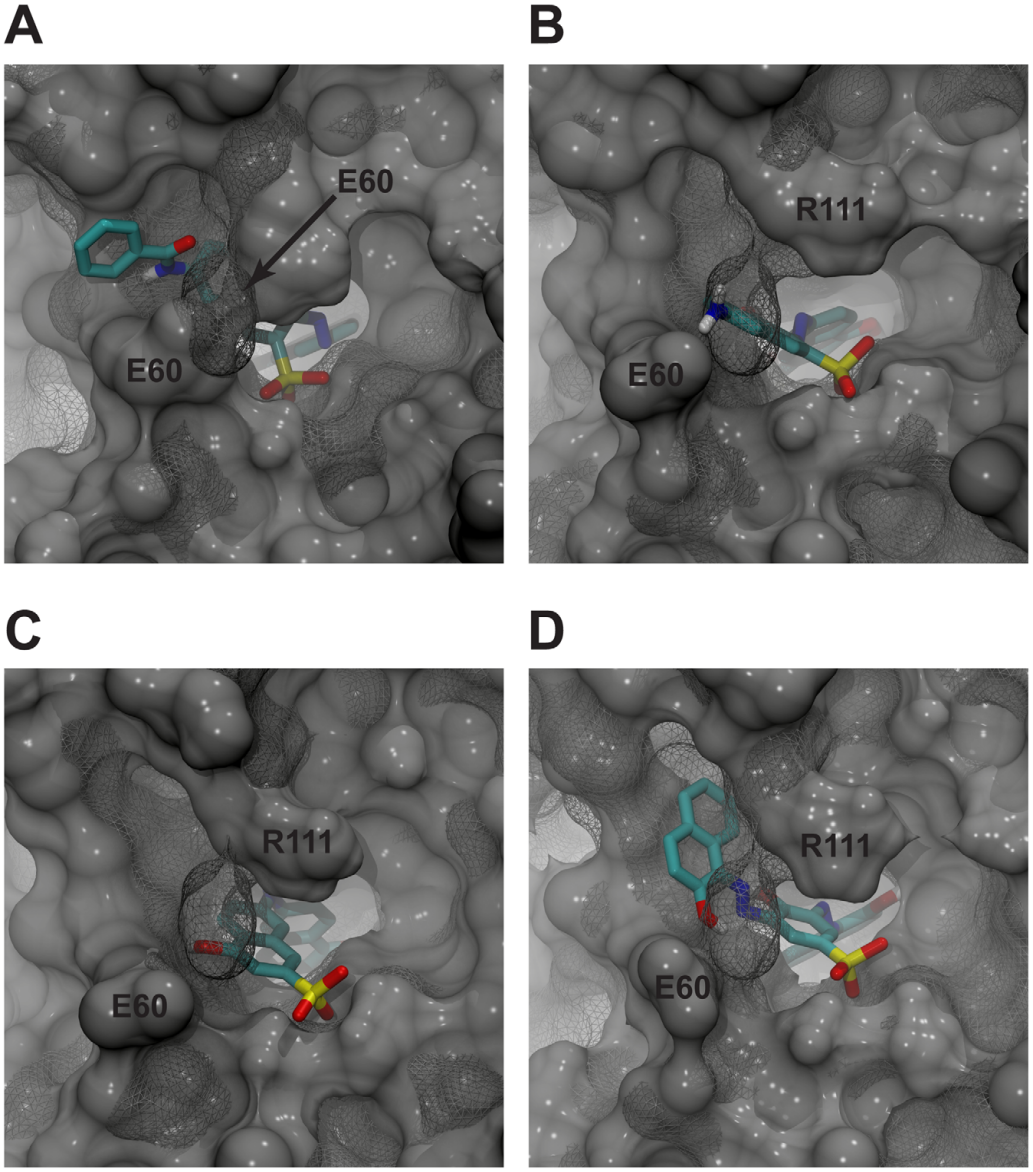
Predicted binding of the identified inhibitors to the respective optimal protein conformation from the ensemble. The *Tb*REL1 crystal structure is shown in mesh. The E60 and R111 residues of the optimal conformations are labeled directly, and the E60 residue of the crystal structure is labeled with an arrow. In all four cases, the closed position of the crystal-structure E60 residue would have prevented optimal ligand binding. A) V1. B) V2. C) V3. D) V4.

In order to investigate why binding to the crystal structure was suboptimal, the crystal structure was compared to the optimal receptor conformation for each of the four ligands. By aligning the best-scoring MD-generated receptor structures to the crystal structure and visualizing both proteins and ligands, it is evident that in all four cases the crystallographic position of E60 prevented optimal binding. During the molecular dynamics simulation, however, E60 extends its contact with R111 (initial contact distance 5.35 Å; final interaction distance greater than 11 Å). This movement opens a wide cleft that is favorably occupied by all four of the novel inhibitors (Figure 3). This unique binding mode, described in more detail below, would not have been identified had protein-receptor flexibility been ignored.

### RMSD Clustering Provides a Population-Based Structural Analysis

To further explore the role that receptor flexibility plays in inhibitor binding, we grouped the frames of the MD trajectory into sets of geometrically similar conformations using an RMSD-based clustering algorithm. Each cluster contains a central structure, or centroid, whose structural characteristics and binding properties are representative of all cluster members. Similar to QR factorization [14], [20], RMSD clustering reduces the MD ensemble to a representative set of (centroid) conformations. However, unlike QR factorization, RMSD clustering provides an approximate idea of the probability of sampling a set of geometrically similar conformations based on the fraction of conformations contained within each cluster [21].

Assuming the conformations sampled along the inhibitor-bound trajectories are similar to those observed during the ATP-bound trajectory, the receptor-inhibitor interactions characteristic of the most populated clusters, which represent the most frequently visited system conformations, should contribute most to ligand affinity. Indeed, the representative protein structure that best accommodates V1, V3, and V4 from the QR-factorization ensemble, as judged by the AutoDock score, belongs to the most populated RMSD-based cluster. The protein conformation that best accommodates V2 belongs to the second most populated RMSD-based cluster.

**Figure 4:**
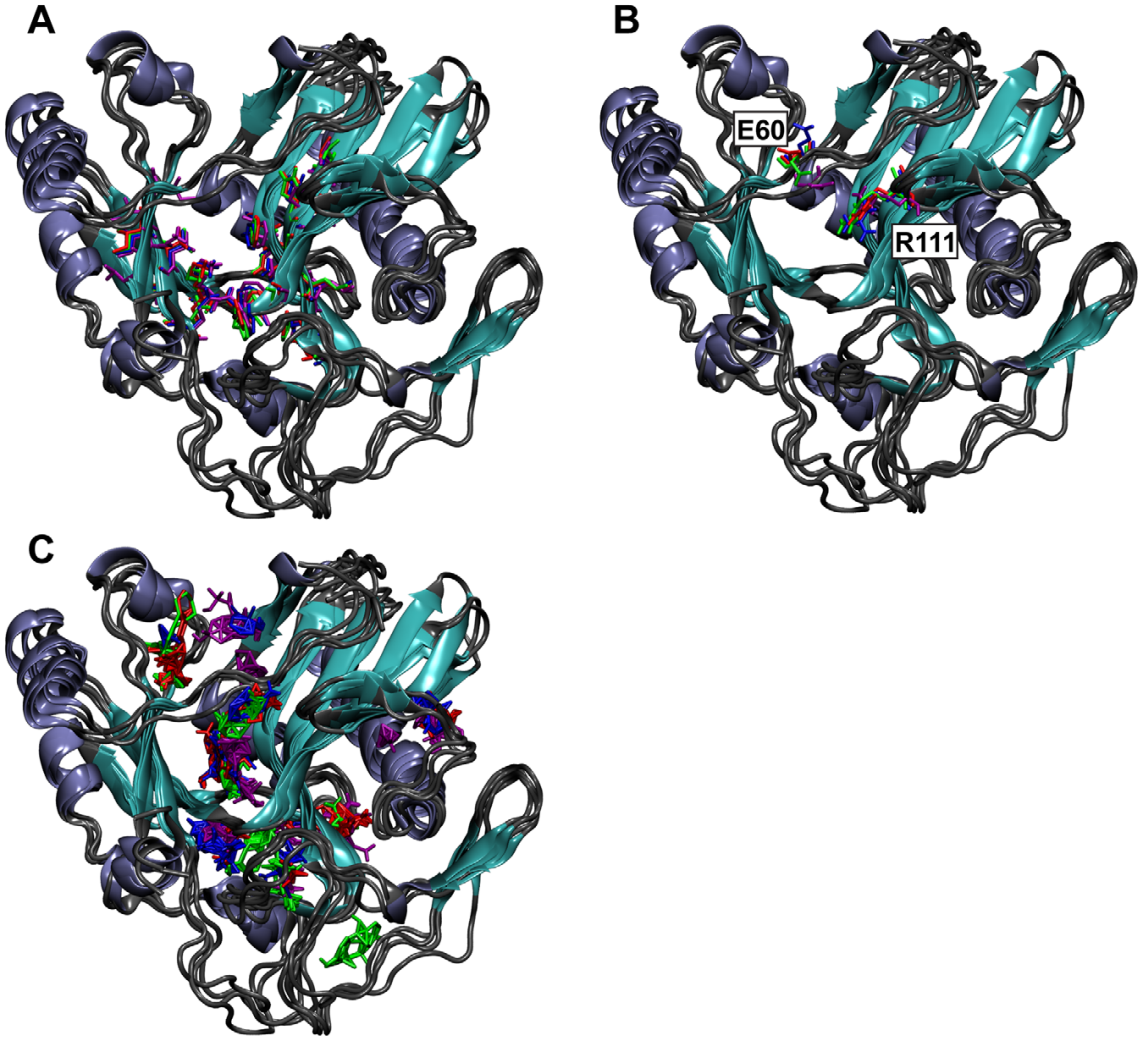
The centroids of the three most populated RMSD clusters aligned to the *Tb*REL1 crystal structure. Coils are colored gray, sheets are colored cyan, and helices are colored ice blue. A) Residues lining the deep end of the binding pocket used for RMSD clustering are shown in a licorice graphical representation. Residues from the centroid of the first, second, and third most populated clusters are shown in red, blue, and green, respectively. Residues from the crystal structure are shown in purple. B) E60 and R111 conformational variability. Coloring is as in A). The orientation of E60 in the centroids of the top two most populated clusters differs from that of the third cluster, as well as from that of the crystal structure. C) Consensus binding sites of the organic solvent probes used in the computational fragment mapping analysis. Solvent probe clusters are colored to match the coloring in A) and B).

The conformations sampled by the MD trajectory were grouped into 8 clusters when an RMSD similarity cutoff of 0.085 Å was used; 93.5% of the trajectory conformations were contained in the three most populated clusters. The conformational variability among the centroids of the top three clusters suggests two dynamically distinct active-site regions. Deep within the inhibitor-binding cleft, where F209 forms π-π stacking interactions with the sulfonated naphthalene moiety of each inhibitor, the conformational differences among the centroids are modest, consisting of only subtle amino-acid side-chain shifts (Figure 4A). Given the rigidity of this region and the similarity between naphthalene and the adenosine of ATP, the native *Tb*REL1 substrate, we hypothesize that the naphthalene scaffold is highly complimentary to the modest conformational fluctuations observed at the deep end of the binding pocket.

In contrast, conformational variability at the binding-site periphery near the solvent interface is much larger (Figure 4B). As the predicted binding modes of the validated inhibitors initially suggested, the varied positions of E60 relative to R111 are particularly notable. In the centroid conformation of the first and second most populated clusters, a cleft is again seen between E60, which is directed into bulk solvent, and R111, which is directed toward the inhibitor binding site. The distances between E60(OE2) and R111(NH1) are 9.01 Å and 10.96 Å, respectively. These two open-cleft clusters represent 83% of the entire trajectory. In the centroid conformation of the third most populated cluster, representing **11**% of the entire trajectory, the cleft is narrowed; E60 is directed downward, toward R111, and the distance between E60(OE2) and R111(NH1) is only 7.14 Å, closer to the closed-cleft crystal-structure distance of 5.35 Å. As noted previously, all four novel inhibitors are predicted to occupy this previously uncharacterized cleft, suggesting that it is pharmacologically important.

This new cleft also presents an opportunity to develop compounds with improved specificity over the related human DNA ligases. A structural and sequence alignment of the superfamily members [18] reveals key sequence differences in relative positions between REL1 and human DNA ligase (PDB: 1X9N). In REL1, residues I59-E60-I61-D62 line the newly revealed cleft and make contact with several of the bound inhibitors. In human DNA ligase 1 (PDB 1X9N), the equivalent residues are M543-L544-A545-H546. The strategic design of REL1 inhibitors to take advantage of the variable contacts in this area, particularly the exposed side chains of the residues lining the cleft, may present novel avenues to design compounds with increased selectivity for the trypanosomal enzymes.

### Computational Fragment Mapping

To explore the pharmacological importance of the E60-R111 cleft in greater depth, computational fragment mapping was carried out on both the centroids of the three most populated clusters as well as the crystal structure (Figure 4C). Computational fragment mapping estimates the binding affinity of fragment-sized organic groups and clusters them into consensus-binding regions. These consensus-binding regions (a.k.a. hot spots) represent regions of receptor sites that are the principal contributors to the ligand-binding energy. Importantly, these computationally predicted sites have been shown to correlate well with fragment-binding hot spots as determined *via* biophysical experiments in numerous studies [27], [29], [30].

Fragment mapping confirmed that the *Tb*REL1 active site can be divided into two regions, as two consensus sites were apparent. The first site, conserved among the centroids of the three most populated clusters as well as the crystal structure, is found deep in the inhibitor-binding cleft, where both the adenine of the native ATP substrate and the sulfonated-naphthalene moieties of the novel inhibitors bind. The conservation of this solvent cluster supports the pharmacological importance of this region and is in harmony with the predicted docking poses of the four novel inhibitors.

The second consensus site is found in the previously uncharacterized E60-R111 cleft. Notably, while conserved among the three most populated clusters, this site is entirely absent in the crystal structure, likely because the closed E60-R111 cleft of that structure occludes solvent-probe binding. Naphthalene-based inhibitors docked into the crystal structure are predicted to interact only with the high-affinity region deep in the binding pocket; at the active-site periphery, binding to the high-affinity region in the E60-R111 cleft is impossible, and so the predicted binding affinity is less favorable. Hence, the fragment-mapping approach supports the presence of an additional pharmacologically relevant feature of the ATP binding pocket. It also helps to explain why those compounds eventually confirmed as genuine inhibitors were not initially ranked among the top-scoring candidates.

While fragment mapping did reveal a high-affinity region in the E60-R111 cleft of the centroid representing the third most populated cluster, this region does not extend as far into the cleft as the corresponding clusters of the top two centroids. This fact, together with the narrower cleft width, may partly explain why none of the four novel inhibitors was predicted to bind to receptor conformations of the third most populated cluster.

### Analysis of Predicted Binding Poses

In order to analyze the predicted binding mode of the four confirmed *Tb*REL1 inhibitors, the protein conformation from the ensemble generated by QR-factorization that gave the best AutoDock-predicted binding energy (i.e. the “optimal receptor”) was visualized together with the associated docked ligand. In all cases, the electronegative group at the naphthalene C2 position was buried deep within the active site, forming interactions with R288, as expected. Additionally, three of the four ligands, similar to the three most potent *Tb*REL1 inhibitors identified previously [14], had hydroxyl groups in the naphthalene 4 position, suggesting that the hydrogen bonds formed with E86 and V88 are also critical to ligand binding (Figure S2, upper rows). A fourth ligand, V4, had a hydroxyl group in the naphthalene 6 position, were it could form hydrogen bonds with the backbone carbonyl oxygen atom of V88 and the side-chain amino group of K87.

At the active-site periphery, all four of the confirmed inhibitors had secondary sulfonate groups that docked near the more positively charged side of the active-site periphery, opposite the R111 residue (Figure S3), where they interact with K307, R309, and K87 (Figure S2, bottom rows). In contrast, the peripheral, negatively charged sulfonate groups of previous NDS inhibitors, substituents of the naphthalene core itself, interacted principally with R111. The new inhibitors do not entirely neglect R111, however; all four compounds are predicted to participate in π-cation interactions with this residue.

In addition to these electrostatic interactions, the four novel inhibitors are predicted to interact with other protein residues at the active-site periphery (Figure S2, bottom rows). In some ways, these interactions mimic the interactions between *Tb*REL1 and its native substrate, ATP. V1 forms a hydrogen bond with the R111 guanidinium group, similar to the bond formed between R111 and the ATP gamma phosphate. V1 also forms a hydrogen bond with the E159 side-chain carboxylate group, similar to the bond formed with the ATP 2′ ribose hydroxyl group. V1 forms unique interactions with *Tb*REL1 as compared to the substrate; V1 forms a hydrogen bond with the backbone carbonyl of Y58, a residue that does not participate in ATP binding (Figure S2A).

V2 is predicted to participate in only one hydrogen bond at the active-site periphery. This bond is formed with the E60 side-chain carboxylate group, a group that does not participate in ATP binding (Figure S2B). V3 and V4 are likewise predicted to form only one hydrogen bond at the active-site periphery, a bond with the I59 backbone carbonyl. This same backbone carbonyl forms a hydrogen bond with the 3′ hydroxyl group of the ATP ribose (Figure S2C).

### Conclusion/Future Directions

Unfortunately, first-stage HAT treatments such as pentamidine and suramin have harsh side effects [3], and second-stage treatments such as melarsoprol can be fatal. The pharmaceutical industry has been slow to develop novel trypanocidal therapeutics because HAT infections occur primarily in developing countries with little market appeal; indeed, the only novel trypanocidal therapeutic registered in the last 50 years is eflornithine [43], a drug that is likely only available because it can also be sold as a topical cosmetic cream for the treatment of hirsutism in developed countries.

Given the hesitancy of the pharmaceutical industry, in recent years academia has played an increasing role in HAT drug-discovery efforts (e.g. [44]). Amaro et al. recently identified inhibitors based on a 4,5-dihydroxynaphthalene-2,7-disulfonate scaffold that target *T. brucei* RNA editing ligase 1 (*Tb*REL1), a validated drug target in these organisms [12]. Unfortunately, these inhibitors, while effective against the *Tb*REL1 protein, were ineffective in whole-cell assays. As Schrodinger's LigPrep software [45] suggested that at pH 7.0 the sulfonates of these compounds are negatively charged, we hypothesize that they are too hydrophilic to cross cellular and organellar *T.*-*brucei* lipid membranes and thus cannot reach their physiological target. The ALogP values of Amaro's S5, V1, and S1 compounds were −1.043, −0.292, and −0.778, respectively (Discovery Studio, Accelrys), likewise suggesting excessive hydrophilicity. Indeed, two of these three compounds, S5 and S1, are too hydrophilic to be considered druglike [46].

Building on the previous work of Amaro et al., we have developed additional *Tb*REL1 inhibitors based on novel naphthalene scaffolds. The compounds proposed in the current work are also sulfonated naphthalenes; however, some of them are more hydrophobic than the naphthalene-based inhibitors identified previously. The ALogP values of V1, V2, V3, and V4 are 0.492, −1.039, −1.112, and 1.835, respectively (Discovery Studio, Accelrys), suggesting that two of the novel inhibitors, V1 and V4, may even prefer a lipid environment. Indeed, V4 was effective against cultured *T. brucei* with an EC_50_ of 2.16 µM (Table 1). To what extent this trypanocidal effect can be attributed to inhibition of REL1 is currently under investigation.

The hydrophobicity and specificity of these compounds, and their ability to reach the mitochondrial matrix, could be further improved by eliminating the charged sulfonate groups. In the virtual screen presented here, naphthalenes with carboxylic acids and nitro groups were included to see if the sulfonate groups could be replaced with less electronegative functional groups. Unfortunately, none of the compounds with carboxylate groups scored well enough to justify experimental testing, and the few nitro-group containing compounds that were tested failed to inhibit *Tb*REL1. Rather than replacing the sulfonate groups, a better strategy may therefore be to modify those groups in order to neutralize their charge. For example, replacing the sulfonate groups with sulfonamides, a similar functional group that is not charged, may decrease hydrophilicity while preserving important protein-ligand interactions.

Both molecular docking and computational fragment mapping indicate that a new cleft revealed by the molecular dynamics simulations may play a role in the favorable binding of these four novel *Tb*REL1 inhibitors. Furthermore, RMSD-based clustering indicated that this previously uncharacterized cleft persists for a majority of the MD trajectory.

In the future, further drug optimization is needed. Three of the four novel compounds contain diazene linkers that may be hydrolysable *in vivo*. Furthermore, the nitrogen atoms of these linkers are not predicted to participate in hydrogen bonds with the protein; replacing one or both of them with carbon atoms may therefore decrease hydrophilicity without sacrificing compound potency. Additionally, some of the compounds contain other moieties like hydroxyl and amino groups that are not predicted to contribute to inhibitor binding. Perhaps these groups could likewise be eliminated.

## Supporting Information

Figure S1 The sixteen fragments used in the computational fragment mapping.
(8.66 MB TIF)

Figure S2 *Tb*REL1 binding. The top rows show binding deep within the active site, and the bottom rows show binding at the active-site periphery. Solid black lines represent hydrogen bonds. Electropositive residues at the active-site periphery are highlighted in yellow. The carbons of the F209 phenyl ring are shown in licorice. Portions of the protein were removed to improve clarity. A) The predicted binding pose of V1. B) The predicted binding pose of V2. C) The predicted binding pose of V3. D) The predicted binding pose of V4.
(7.23 MB TIF)

Figure S3 The *Tb*REL1 active-site periphery. Positively charged residues are highlighted in yellow. A) The predicted position of the NDS peripheral sulfonate. B) The predicted position of the peripheral sulfonates of V1, V2, V3, and V4.
(8.61 MB TIF)

Table S1 The twelve compounds that were tested experimentally.
(0.20 MB DOC)
