# Supplementary figures and images for: Novel Naphthalene-Based Inhibitors of Trypanosoma brucei RNA Editing Ligase 1
Source: PLoS Negl Trop Dis. 2010 Aug 24;4(8):e803. doi: 10.1371/journal.pntd.0000803 (PMC2927429; doi:10.1371/journal.pntd.0000803)

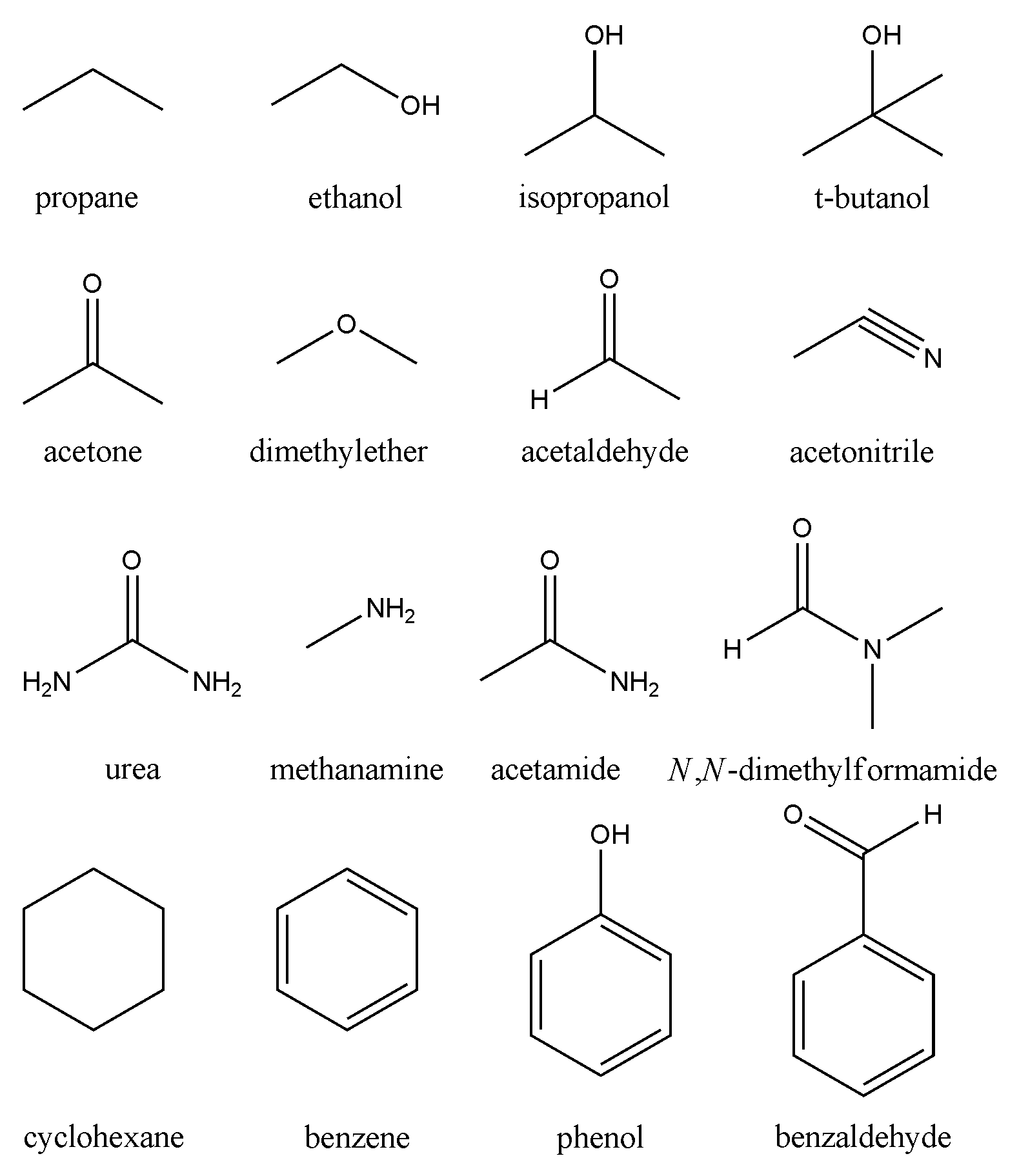

Supplement: Figure S1 — The sixteen fragments used in the computational fragment mapping. (8.66 MB TIF) [file pntd.0000803.s001.tif]

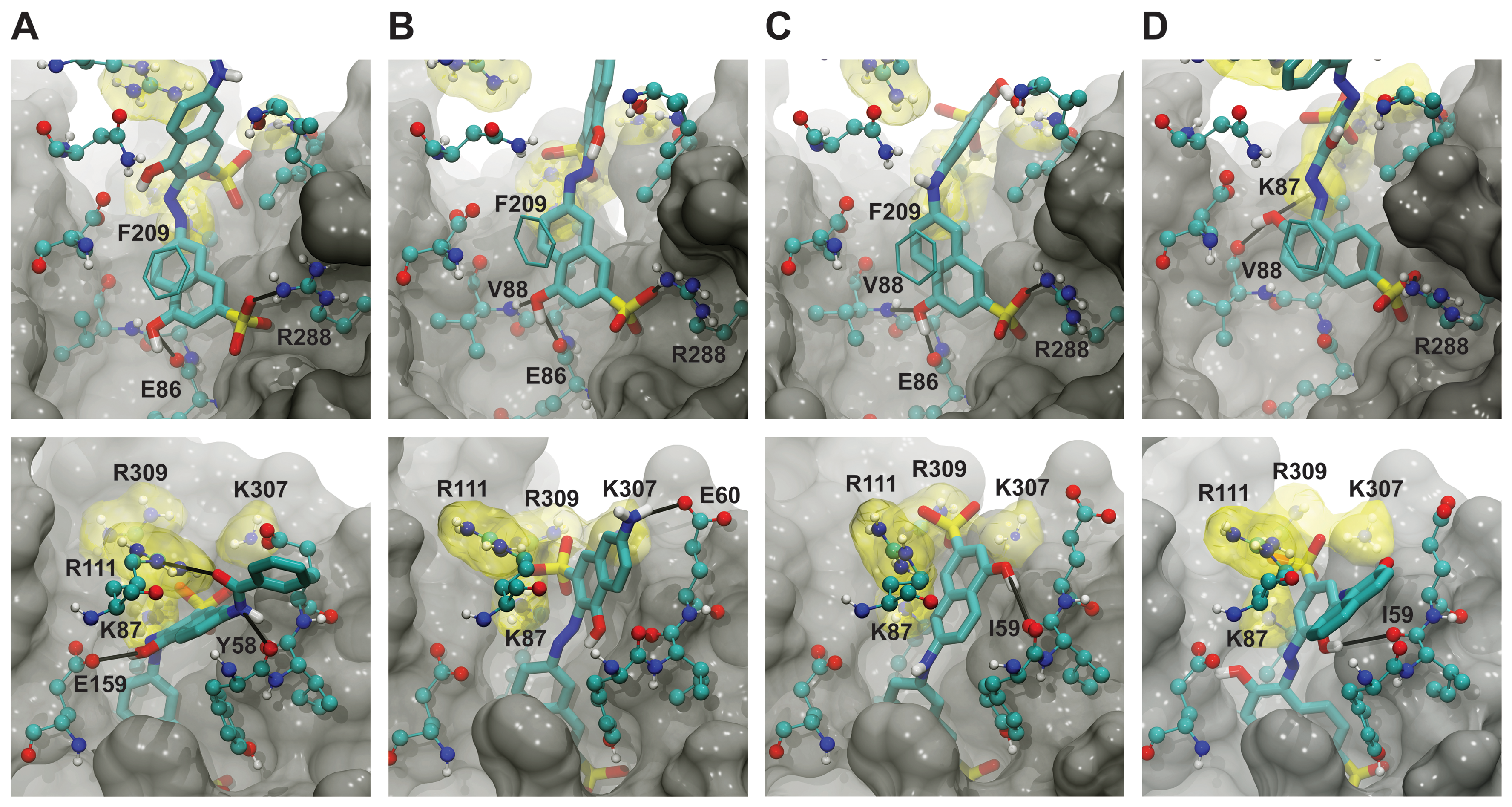

Supplement: Figure S2 — TbREL1 binding. The top rows show binding deep within the active site, and the bottom rows show binding at the active-site periphery. Solid black lines represent hydrogen bonds. Electropositive residues at the active-site periphery are highlighted in yellow. The carbons of the F209 phenyl ring are shown in licorice. Portions of the protein were removed to improve clarity. A) The predicted binding pose of V1. B) The predicted binding pose of V2. C) The predicted binding pose of V3. D) The predicted binding pose of V4. (7.23 MB TIF) [file pntd.0000803.s002.tif]

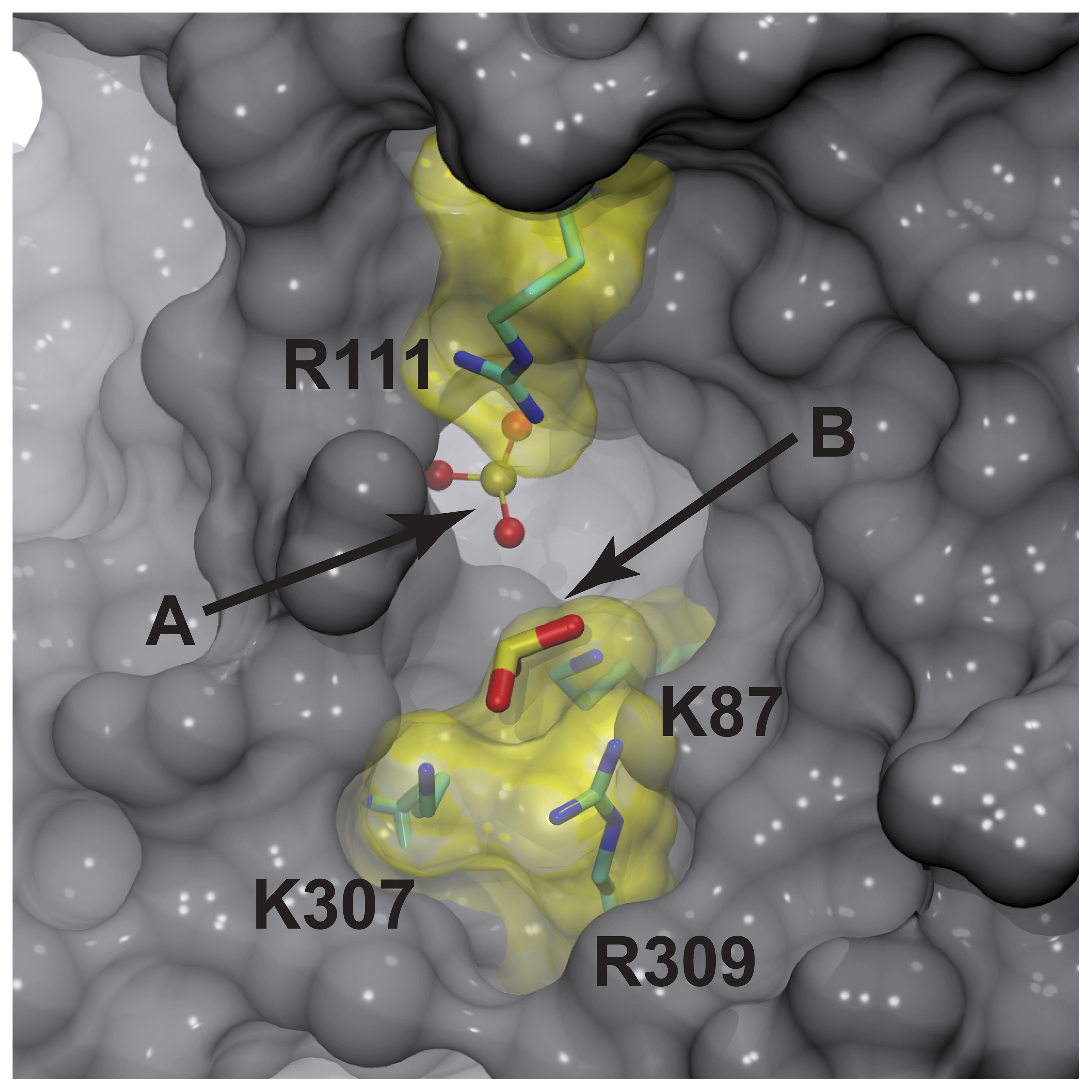

Supplement: Figure S3 — The TbREL1 active-site periphery. Positively charged residues are highlighted in yellow. A) The predicted position of the NDS peripheral sulfonate. B) The predicted position of the peripheral sulfonates of V1, V2, V3, and V4. (8.61 MB TIF) [file pntd.0000803.s003.tif]
