# Supplementary material for: Novel Naphthalene-Based Inhibitors of Trypanosoma brucei RNA Editing Ligase 1
Source: PLoS Negl Trop Dis. 2010 Aug 24;4(8):e803. doi: 10.1371/journal.pntd.0000803 (PMC2927429; doi:10.1371/journal.pntd.0000803)
Supplement: Table S1 — The twelve compounds that were tested experimentally. (0.20 MB DOC) [file pntd.0000803.s004.doc]

|  | ID / Source | Predicted Binding Energy (kcal/mol)  (± Standard Deviation) | Structure | IC_50_ (μM) |
| --- | --- | --- | --- | --- |
| 1 | V4  Sigma Mordant Black 25 | -12.75 ± 1.61 |  | 1.59 ± 1.10 |
| 2 | NSC42067 | -12.46 ± 1.52 |  | 10 - 100 |
| 3 | V2  NSC162535 | -11.93 ± 1.40 |  | 1.53 ± 1.17 |
| 4 | V1  NSC45609 | -11.86 ± 1.39 |  | 2.16 ± 1.20 |
| 5 | ChemBridge 5303429 | -11.07 ± 1.48 |  | > 100 |
| 6 | ChemBridge 5250272 | -11.05 ± 0.55 |  | > 100 |
| 7 | ChemBridge 5250329 | -10.82 ± 0.50 |  | > 100 |
| 8 | Sigma Acid Black 26 | -10.54 ± 1.30 |  | > 100 |
| 9 | NSC8674 | -10.53 ± 1.04 | 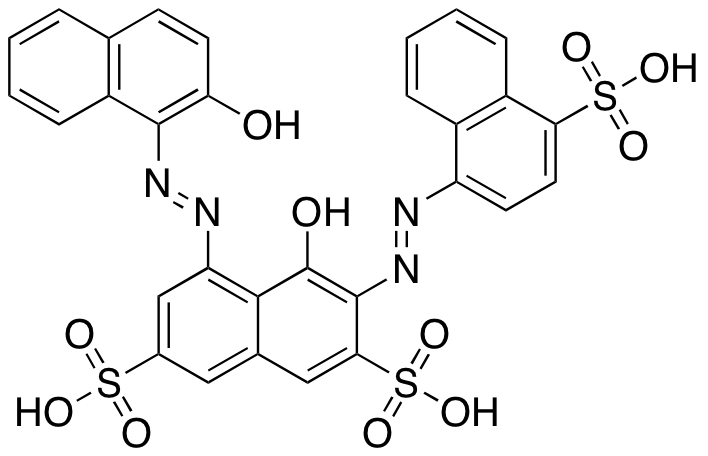 | 10 - 100 |
| 10 | NSC45577 | -10.51 ± 1.20 |  | 10 - 100 |
| 11 | NSC75908 | -10.29 ± 0.79 |  | 10 - 100 |
| 12 | V3  NSC1698 | -10.23 ± 0.98 |  | 8.36 ± 1.71 |
